# No correlation between carotid intima-media thickness and long-term glycemic control in individuals with type 1 diabetes

**DOI:** 10.1007/s00592-023-02211-y

**Published:** 2023-12-10

**Authors:** Jussi Inkeri, Valma Harjutsalo, Juha Martola, Jukka Putaala, Per-Henrik Groop, Daniel Gordin, Lena M. Thorn, the FinnDiane Study Group, S. Koivula, T. Uggeldahl, T. Forslund, A. Halonen, A. Koistinen, P. Koskiaho, M. Laukkanen, J. Saltevo, M. Tiihonen, SM. Forsen, H. Granlund, A.-C. Jonsson, B. Nyroos, P. Kinnunen, A. Orvola, T. Salonen, A. Vähänen, R. Paldanius, M. Riihelä, L. Ryysy, H. Laukkanen, P. Nyländen, A. Sademies, S. Anderson, B. Asplund, U. Byskata, P. Liedes, M. Kuusela, T. Virkkala, A. Nikkola, E. Ritola, M. Niska, H. Saarinen, E. Oukko-Ruponen, T. Virtanen, A. Lyytinen, H. Kari, T. Simonen, A. Kaprio, J. Kärkkäinen, B. Rantaeskola, P. Kääriäinen, J. Haaga, A.-L. Pietiläinen, S. Klemetti, T. Nyandoto, E. Rontu, S. Satuli-Autere, R. Toivonen, H. Virtanen, R. Ahonen, M. Ivaska-Suomela, A. Jauhiainen, M. Laine, T. Pellonpää, R. Puranen, A. Airas, J. Laakso, K. Rautavaara, M. Erola, E. Jatkola, R. Lönnblad, A. Malm, J. Mäkelä, E. Rautamo, P. Hentunen, J. Lagerstam, M. Feodoroff, D. Gordin, O. Heikkilä, K. Hietala, J. Fagerudd, M. Korolainen, L. Kyllönen, J. Kytö, S. Lindh, K. Pettersson-Fernholm, M. Rosengård-Bärlund, A. Sandelin, L. Thorn, J. Tuomikangas, T. Vesisenaho, J. Wadén, V. Sipilä, T. Kalliomäki, J. Koskelainen, R. Nikkanen, N. Savolainen, H. Sulonen, E. Valtonen, L. Norvio, A. Hämäläinen, E. Toivanen, A. Parta, I. Pirttiniemi, S. Aranko, S. Ervasti, R. Kauppinen-Mäkelin, A. Kuusisto, T. Leppälä, K. Nikkilä, L. Pekkonen, K. Nuorva, M. Tiihonen, S. Jokelainen, K. Kananen, M. Karjalainen, P. Kemppainen, A.-M. Mankinen, A. Reponen, M. Sankari, H. Stuckey, P. Suominen, A. Lappalainen, M. Liimatainen, J. Santaholma, A. Aimolahti, E. Huovinen, V. Ilkka, M. Lehtimäki, E. Pälikkö-Kontinen, A. Vanhanen, E. Koskinen, T. Siitonen, E. Huttunen, R. Ikäheimo, P. Karhapää, P. Kekäläinen, M. Laakso, T. Lakka, E. Lampainen, L. Moilanen, S. Tanskanen, L. Niskanen, U. Tuovinen, I. Vauhkonen, E. Voutilainen, T. Kääriäinen, E. Isopoussu, E. Kilkki, I. Koskinen, L. Riihelä, T. Meriläinen, P. Poukka, R. Savolainen, N. Uhlenius, A. Mäkelä, M. Tanner, L. Hyvärinen, K. Lampela, S. Pöykkö, T. Rompasaari, S. Severinkangas, T. Tulokas, P. Erola, L. Härkönen, P. Linkola, T. Pekkanen, I. Pulli, E. Repo, T. Granlund, K. Hietanen, M. Porrassalmi, M. Saari, T. Salonen, M. Tiikkainen, I.-M. Jousmaa, J. Rinne, A. Mäkelä, P. Eloranta, H. Lanki, S. Moilanen, M. Tilly-Kiesi, A. Gynther, R. Manninen, P. Nironen, M. Salminen, T. Vänttinen, I. Pirttiniemi, A.-M. Hänninen, U.-M. Henttula, P. Kekäläinen, M. Pietarinen, A. Rissanen, M. Voutilainen, A. Burgos, K. Urtamo, E. Jokelainen, P.-L. Jylkkä, E. Kaarlela, J. Vuolaspuro, L. Hiltunen, R. Häkkinen, S. Keinänen-Kiukaanniemi, R. Ikäheimo, H. Haapamäki, A. Helanterä, S. Hämäläinen, V. Ilvesmäki, H. Miettinen, P. Sopanen, L. Welling, V. Sevtsenko, M. Tamminen, M.-L. Holmbäck, B. Isomaa, L. Sarelin, P. Ahonen, P. Merisalo, E. Muurinen, K. Sävelä, M. Kallio, B. Rask, S. Rämö, A. Holma, M. Honkala, A. Tuomivaara, R. Vainionpää, K. Laine, K. Saarinen, T. Salminen, P. Aalto, E. Immonen, L. Juurinen, A. Alanko, J. Lapinleimu, P. Rautio, M. Virtanen, M. Asola, M. Juhola, P. Kunelius, M.-L. Lahdenmäki, P. Pääkkönen, M. Rautavirta, T. Pulli, P. Sallinen, M. Taskinen, E. Tolvanen, T. Tuominen, H. Valtonen, A. Vartia, S.-L. Viitanen, O. Antila, E. Korpi-Hyövälti, T. Latvala, E. Leijala, T. Leikkari, M. Punkari, N. Rantamäki, H. Vähävuori, T. Ensala, E. Hussi, R. Härkönen, U. Nyholm, J. Toivanen, A. Vaden, P. Alarotu, E. Kujansuu, H. Kirkkopelto-Jokinen, M. Helin, S. Gummerus, L. Calonius, T. Niskanen, T. Kaitala, T. Vatanen, P. Hannula, I. Ala-Houhala, R. Kannisto, T. Kuningas, P. Lampinen, M. Määttä, H. Oksala, T. Oksanen, A. Putila, H. Saha, K. Salonen, H. Tauriainen, S. Tulokas, T. Kivelä, L. Petlin, L. Savolainen, A. Artukka, I. Hämäläinen, L. Lehtinen, E. Pyysalo, H. Virtamo, M. Viinikkala, M. Vähätalo, K. Breitholz, R. Eskola, K. Metsärinne, U. Pietilä, P. Saarinen, R. Tuominen, S. Äyräpää, K. Mäkinen, P. Sopanen, S. Ojanen, E. Valtonen, H. Ylönen, M. Rautiainen, T. Immonen, I. Isomäki, R. Kroneld, L. Mustaniemi, M. Tapiolinna-Mäkelä, S. Bergkulla, U. Hautamäki, V.-A. Myllyniemi, I. Rusk

**Affiliations:** 1 Radiology, HUS Diagnostic Center, University of Helsinki and Helsinki University Hospital, Helsinki, Finland; 2 Folkhälsan Research Center, Biomedicum Helsinki, University of Helsinki, P.O. Box 63 (C318b), 00014 Helsinki, Finland; 3 Minerva Foundation Institute for Medical Research, Helsinki, Finland; 4 Research Program for Clinical and Molecular Metabolism, University of Helsinki, Helsinki, Finland; 5 Department of Nephrology, University of Helsinki and Helsinki University Hospital, Helsinki, Finland; 6 Department of Clinical Neuroscience, Karolinska Institute, Stockholm, Sweden; 7 Neurology, University of Helsinki and Helsinki University Hospital, Helsinki, Finland; 8 Department of Diabetes, Central Clinical School, Monash University, Melbourne, Australia; 9 Department of General Practice and Primary Health Care, University of Helsinki and Helsinki University Hospital, Helsinki, Finland

**Keywords:** Type 1 diabetes, Carotid intima-media thickness, Glycemic control, Cardiovascular disease

## Abstract

**Aims:**

To determine whether carotid intima-media thickness (CIMT), a surrogate marker of cardiovascular disease (CVD), is associated with long-term blood glucose control in individuals with type 1 diabetes (T1D).

**Methods:**

We recruited 508 individuals (43.4% men; median age 46.1, IQR 37.8–55.9 years) with T1D (median diabetes duration of 30.4, IQR 21.2–40.8 years) in a cross-sectional retrospective sub-study, part of the Finnish Diabetic Nephropathy (FinnDiane) Study. Glycated hemoglobin (HbA_1c_) data were collected retrospectively over the course of ten years (HbA_1c_-mean_overall_) prior to the clinical study visit that included a clinical examination, biochemical sampling, and ultrasound of the common carotid arteries.

**Results:**

Individuals with T1D had a median CIMT of 606 μm (IQR 538–683 μm) and HbA_1c_ of 8.0% (7.3–8.8%) during the study visit and HbA_1c_-mean_overall_ of 8.0% (IQR 7.3–8.8%). CIMT did not correlate with HbA_1c_ (*p* = 0.228) at visit or HbA_1c_-mean_overall_ (*p* = 0.063). After controlling for relevant factors in multivariable linear regression analysis, only age was associated with CIMT (*p* < 0.001). After further dividing CIMT into quartiles, no correlation between long-term glucose control and CIMT (%, 1^st^ 8.1 [IQR 7.2–8.9] vs 4^th^ 7.9 [7.4–8.7], *p* = 0.730) was found.

**Conclusions:**

We observed no correlation between long-term blood glucose control and CIMT in individuals with T1D. This finding suggests that the development of early signs of macrovascular atherosclerosis is not strongly affected by the glycemic control in people with T1D.

**Supplementary Information:**

The online version contains supplementary material available at 10.1007/s00592-023-02211-y.

## Introduction

Carotid intima-media thickness (CIMT), a surrogate marker of atherosclerosis, predicts cardiovascular events, particularly myocardial infarction, and stroke. CIMT can be measured using ultrasound of the two innermost layers of the carotid artery wall, tunica intima and tunica media. Ultrasound of the CIMT provides an easy, reproducible non-invasive tool to assess the risk of cardiovascular disease (CVD). It can be measured by a trained technician with a mobile ultrasound device in an outpatient setting [1].

Type 1 diabetes (T1D) increases the risk of CVD [2], and individuals with T1D have a sixfold increased risk of stroke compared to non-diabetic individuals [3]. It is of note that these T1D individuals have increased CIMT compared to healthy controls [1, 4]. We recently demonstrated that CIMT is a potential indicator of cerebral small vessel disease in individuals with T1D, regardless of blood glucose control [5].

Surprisingly, data on the effect of blood glucose control on CIMT in T1D are short. The Epidemiology of Diabetes Interventions and Complications (EDIC) trial, a long-term follow-up of the Diabetes Control and Complications Trial (DCCT), demonstrated that intensive blood glucose treatment of T1D had slowed the progression of CIMT as observed 6 years after the end of the intervention compared to the conventional blood glucose treatment. [6, 7] CIMT correlated with the age and duration of diabetes in a cross-sectional study in adolescents and young adult individuals with T1D, regardless of other clinical covariates. Furthermore, there was a non-significant trend between sex and blood glycated hemoglobin (HbA_1c_) and CIMT. [8] Larsen and colleagues observed an independent association between HbA_1c_ and CIMT in women; however, no correlation was seen among men [9].

We aimed to explore the relationship between CIMT and the current glycemic control, as well as the long-term glycemic control over the preceding ten years, in a well-characterized cohort of Finnish individuals with T1D. The findings of our study may shed light on the underlying metabolic mechanisms at play, potentially building rationales for future research in the field.

## Methods

### Study population

This research was conducted as part of the Finnish Diabetic Nephropathy (FinnDiane) Study, a comprehensive, nationwide, multicenter study that aims to uncover genetic, environmental, and clinical risk factors for micro- and macrovascular complications of T1D. The final aim of the FinnDiane study is to understand underlying causes of diabetes-related complications and to identify potential targets for intervention and prevention. The FinnDiane study protocol has been published previously [10].

For this substudy, a total of 508 individuals with T1D with an onset of diabetes < 40 years and age span ranging from 19.5 to 80.8 years were included. All participants were consecutively enrolled at the FinnDiane Study Center at the Helsinki University Hospital between 2009 and 2019.

### Ethical considerations

The study was carried out in accordance with the Declaration of Helsinki and approved by the Ethics Committee of the Helsinki and Uusimaa Hospital District. Each participant signed a written informed consent.

### Laboratory tests and clinical examination

Blood samples were drawn for the determination of serum lipids and lipoproteins (total cholesterol, high-density lipoprotein [HDL] cholesterol and triglycerides), serum creatinine, HbA_1c_, and high-sensitivity C-reactive protein (hs-CRP). The Friedewald equation was used to calculate the low-density lipoprotein (LDL) cholesterol concentration [11]. The CKD-EPI-formula was used to calculate the estimated glomerular filtration rate (eGFR) [12]. Hypertension was defined as office measurement of systolic blood pressure (SBP) ≥ 140, diastolic blood pressure (DBP) ≥ 90, or the use of anti-hypertensive medication.

Albuminuria was defined by the urinary albumin excretion rate (UAER) ≥ 20 μg/min or UAER ≥ 30 mg/24 h in two out of three consecutive overnight or 24-h urine collections. Individuals (n = 57) on kidney replacement therapy (dialysis or kidney transplant) were included in study but excluded from the albuminuria analysis. Of the individuals, 32 had missing albuminuria data.

Medical records and in-depth questionnaires, previously described, were part of the clinical data [13]. Smoking was defined as current or history of smoking at least one cigarette per day for at least one year. History of retinal photocoagulation was used as a marker of severe diabetic retinopathy. Coronary heart disease was defined as the diagnosis of myocardial infarction or history of coronary revascularization. Stroke was defined as either cerebral infarction or intracerebral hemorrhage. Peripheral vascular disease was defined as history of revascularization of a peripheral artery or lower limb amputation. In this study, cardiovascular events included the diagnosis of myocardial infarction, the need for coronary revascularization, stroke, or peripheral vascular disease.

### Measures of glycemic control

For current glycemic control, we used the level of HbA_1c_, a biomarker that indicates the average blood glucose control over a period of two to three months [14]. HbA_1c_ was measured using standardized assays in a central laboratory (Medix Laboratories, Espoo Finland).

To gain a broader picture of long-term glucose control, we obtained at least five HbA_1c_ values for each individual over a period of ten years preceding the study visit. These values were used to calculate the overall mean HbA_1c_ (HbA_1c_-mean_overall_). These HbA_1c_ values were collected from the medical files and had been determined by standardized methods, high-performance liquid chromatography (HPLC), with a reference range of 4–6% (20–42 mmol/mol).

### Measurement of carotid intima-media thickness

Ultrasound imaging of the carotid arteries was conducted on both the left and right sides. The distal 1-cm segment of the common carotid artery, immediately preceding the point of origin of the bulb, was scanned by a trained nurse, using a specialized ultrasound scanner (MyLab 70, Esaote, Genova, Italy) equipped with a 10-MHz linear probe. The mean of two measurements of the left and right CIMT was calculated. [6, 15]

The scanner was integrated with a radiofrequency-based tracking of the arterial wall (QIMT®), which enables semi-automatic and real-time determination of CIMT values during six cardiac cycles of the far-wall CIMT [16, 17].

### Statistics

Statistical analysis was done with IBM SPSS Statistics 27.0 (IBM, Armonk, NY, USA). Kruskal–Wallis tests were used for the nonparametric data presented as medians (interquartile range). The *X*^2^ test was used for categorical variables. Unadjusted linear regression models were used to analyze relationship between CIMT and HbA_1c_, HbA_1c_-mean_overall_, and age. To analyze the relationship between CIMT and HbA_1c_, HbA_1c_-mean_overall_, and clinically relevant risk factors (age, sex, history of cardiovascular event, history of retinal photocoagulation, systolic blood pressure, use of lipid lowering drug, and eGFR) that differed between CIMT quartiles, multivariable linear regression models were built with the CIMT as dependent variable and HbA_1c_ or HbA_1c_-mean_overall,_ and clinically relevant risk factors as independent variables. The threshold for statistical significance was set at *p* < 0.05.

## Results

### Clinical characteristics

Five hundred and eight individuals with T1D were included in this study, and their clinical characteristics are presented in Table 1. Median age was 46.1 (IQR 37.8–55.9) years and 43.3% were men. The diabetes duration was 30.4 (IQR 21.2–40.8) years. Sixty-nine individuals had a history of a cardiovascular event of which 22 had had a stroke. Two hundred and five individuals had had retinal laser photocoagulation. Systolic blood pressure was 134 mmHg (IQR 122–147 mmHg) and diastolic 76 mmHg (IQR 70–83 mmHg). Two hundred and sixty-nine individuals were taking medication for high blood pressure. The HbA_1c_ at the time of the CIMT measurement was 8.0% (7.3–8.8%), (64 mmol/mol [56–72 mmol/mol]), while the HbA_1c_-mean_overall_ (median count 18, IQR 13–28) was 8.0% (IQR 7.3–8.8%), (64 mmol/mol [IQR 57–72 mmol/mol]). The clinical characteristics are presented in Table 1.

**Table 1:** Clinical characteristics of the study population and study population divided into quartiles of carotid intima-media thickness

Clinical characteristics of the study population	
	Individuals with type 1 diabetes *n* = 508
Age, years	46.1 (37.8–55.9)
Male sex, *n* (%)	220 (43.3)
Diabetes duration, years	30.4 (21.2–40.8)
History of smoking, *n* (%)	208 (41.8)
History of cardiovascular event, *n* (%)	69 (13.7)
History of stroke, *n* (%)	22 (4.4)
History of coronary heart disease, *n* (%)	39 (7.7)
History of peripheral vascular disease, *n* (%)	28 (5.6)
History of retinal photocoagulation, *n* (%)	205 (40.4)
Carotid intima-media thickness, μm	606 (538–683)
Body mass index, kg/m^2^	25.8 (23.2–28.8)
Systolic blood pressure, mmHg	134 (122–147)
Diastolic blood pressure, mmHg	76 (70–83)
Anti-hypertensive drug, *n* (%)	269 (53.2)
Renin–angiotensin–aldosterone system blockers, *n* (%)	219 (43.8)
Total cholesterol, mmol/L	4.4 (3.9–5.0)
High-density lipoprotein, mmol/L	1.5 (1.2–1.8)
Low-density lipoprotein, mmol/L	2.3 (1.9–2.8)
Triglycerides, mmol/L	0.9 (0.7–1.4)
Lipid-lowering drug, *n* (%)	210 (41.7)
Albuminuria (excl. end-stage renal disease), *n* (%)	103 (24.6)
Estimated glomerular filtration rate, ml/min/1.73 m^2^	98 (72–111)
High-sensitivity C-reactive protein, mg/l	1.3 (0.6–3.0)
HbA_1c_, %	8.0 (7.3–8.8)
HbA_1c_, mmol/mol	64 (56–72)
HbA_1c_ count within ten years from study visit, *n*	18 (13–28)
HbA_1c_-mean_overall,_ %	8.0 (7.3–8.8)
HbA_1c_-mean_overall,_ mmol/mol	64 (57–72)

Carotid intima-media thickness quartiles					
	1st (300–537 μm) *n* = 127	2nd (538–606 μm) *n* = 128	3rd (607–682 μm) *n* = 127	4th (683–1138 μm) *n* = 126	*p* value
Age, years	34.4 (31.0–41.2)	43.1 (37.6–49.0)	48.5 (41.9–55.9)	58.8 (51.6–65.8)	< 0.001
Male sex, *n* (%)	43 (33.9)	57 (44.5)	56 (44.1)	64 (50.8)	0.055
Diabetes duration, years	22.0 (18.4–28.1)	27.7 (20.5–38.2)	33.0 (22.6–41.8)	40.8 (34.1–46.4)	< 0.001
History of smoking, *n* (%)	50 (39.7)	47 (37.3)	54 (43.5)	57 (46.7)	0.449
Body mass index, kg/m^2^	25.4 (22.7–28.2)	26.1 (23.3–28.9)	26.2 (23.5–30.4)	25.6 (23.2–28.4)	0.318
Systolic blood pressure, mmHg	123 (115–140)	132 (124–147)	136 (125–148)	144 (129–155)	< 0.001
Diastolic blood pressure, mmHg	75 (70–84)	77 (71–84)	76 (69–82)	73 (68–80)	0.039
Anti-hypertensive drug, *n* (%)	45 (35.4)	58 (45.3)	76 (59.8)	90 (72.6)	< 0.001
Renin–angiotensin–aldosterone system blockers, n (%)	35 (28.0)	48 (37.5)	59 (47.6)	77 (62.6)	< 0.001
Total cholesterol, mmol/L	4.4 (4.0–5.1)	4.5 (3.9–5.0)	4.3 (3.8–4.9)	4.4 (3.9–5.1)	0.381
HDL cholesterol, mmol/L	1.5 (1.2–1.8)	1.5 (1.2–1.8)	1.5 (1.2–1.8)	1.6 (1.2–2.1)	0.236
LDL cholesterol, mmol/L	2.4 (2.0–3.0)	2.4 (1.9–2.9)	2.2 (1.8–2.7)	2.3 (1.9–2.7)	0.026
Triglycerides, mmol/L	0.9 (0.7–1.3)	0.9 (0.7–1.3)	1.0 (0.7–1.5)	1.0 (0.7–1.5)	0.390
Lipid-lowering drug, *n* (%)	26 (20.8)	51 (39.8)	66 (52.4)	67 (54.0)	< 0.001
High-sensitivity C-reactive protein, mg/l	1.3 (0.6–2.5)	1.2 (0.5–3.1)	1.3 (0.6–3.3)	1.4 (0.7–3.1)	0.799
Albuminuria (excl. end-stage kidney disease), *n* (%)	20 (16.9)	29 (26.9)	28 (27.7)	26 (28.3)	0.157
Estimated GFR, ml/min/1.73 m^2^	110.8 (96.3–117.1)	102.7 (83.7–114.2)	94.7 (66.5–108.5)	83.7 (62.1–95.6)	< 0.001
History of cardiovascular event, *n* (%)	3 (2.4)	11 (8.7)	21 (16.5)	34 (27.0)	< 0.001
History of stroke, *n* (%)	0 (0.0)	3 (2.4)	6 (4.7)	13 (10.3)	< 0.001
History of coronary heart disease, *n* (%)	1 (0.8)	6 (4.7)	14 (11.1)	18 (14.5)	< 0.001
History of peripheral vascular disease, *n* (%)	2 (1.6)	5 (3.9)	9 (7.1)	12 (9.6)	0.032
History of retinal photocoagulation, *n* (%)	37 (29.1)	47 (36.7)	58 (45.7)	63 (50.0)	0.003
HbA_1c_, %	7.9 (7.2–8.8)	8.0 (7.4–8.7)	8.0 (7.3–8.9)	8.0 (7.2–8.6)	0.962
HbA_1c_, mmol/mol	63 (55–72)	64 (57–72)	64 (56–73)	64 (56–71)	0.959
HbA_1c_ count within ten years from study visit, n	16 (11–26)	20 (13–29)	18 (11–27)	18 (13–29)	0.275
HbA_1c_-mean_overall,_ %	8.1 (7.2–8.9)	8.0 (7.4–8.7)	8.0 (7.4–8.9)	7.9 (7.4–8.7)	0.730
HbA_1c_-mean_overall,_ mmol/mol	65 (56–74)	63 (57–71)	64 (57–74)	63 (58–72)	0.720

Data are median (interquartile range) unless otherwise indicated

### CIMT

**Fig. 1:**
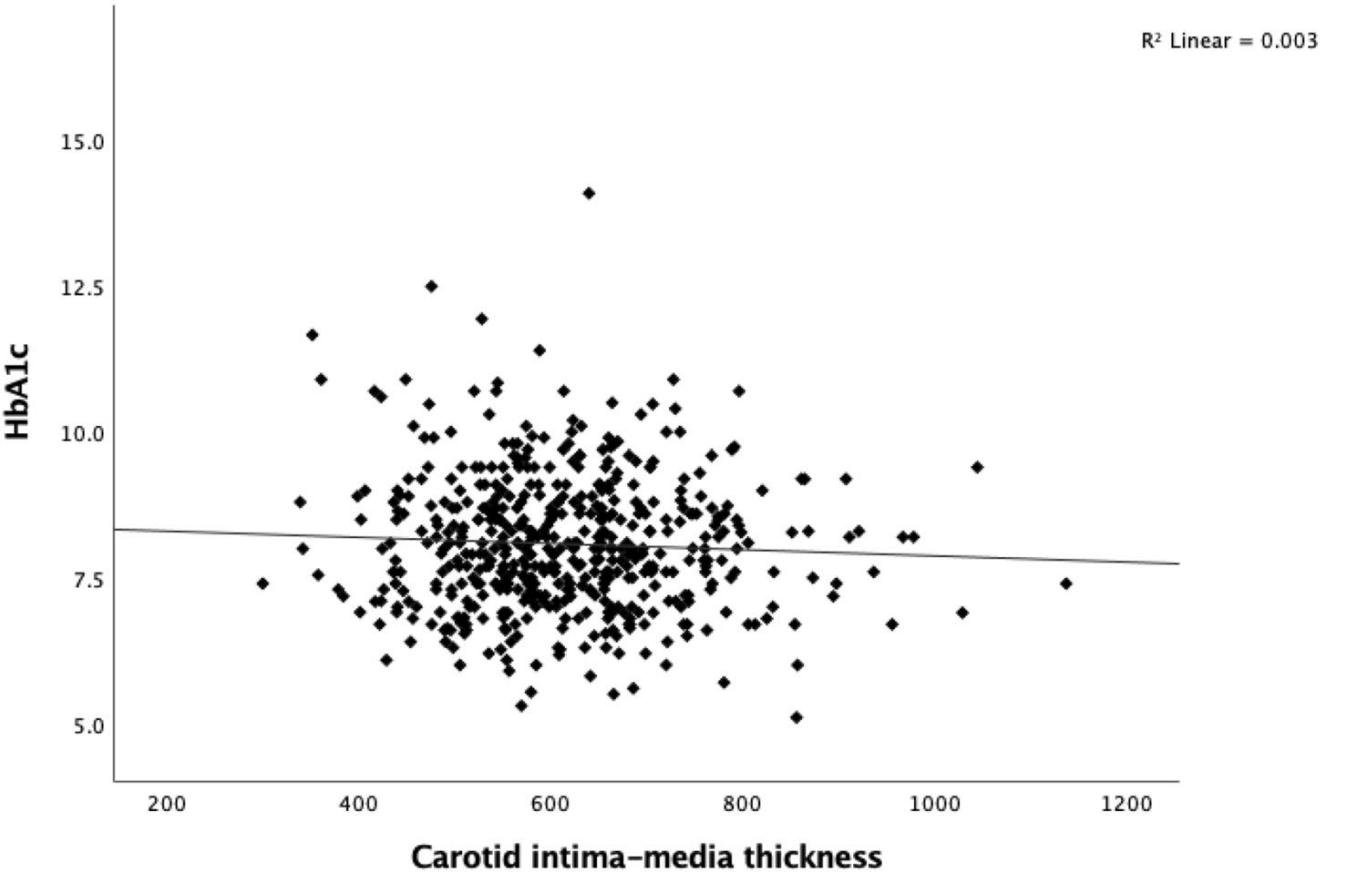
Carotid intima-media thickness (μm) by HbA_1c_ (%) in individuals with type 1 diabetes (*p* = 0.228)

**Fig. 2:**
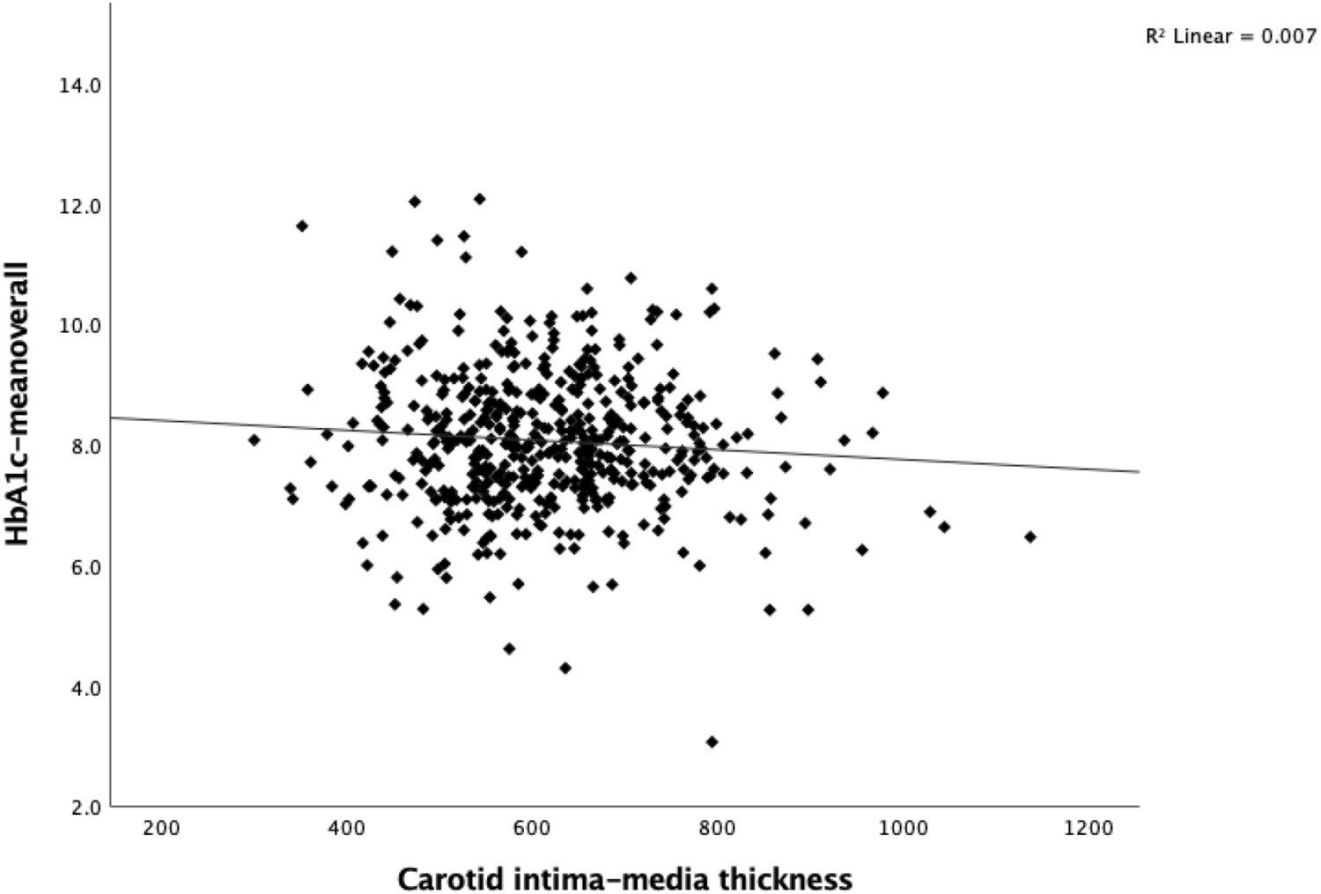
Carotid intima-media thickness (μm) by HbA_1c_-mean_overall_ (%) in individuals with type 1 diabetes (*p* = 0.063)

**Fig. 3:**
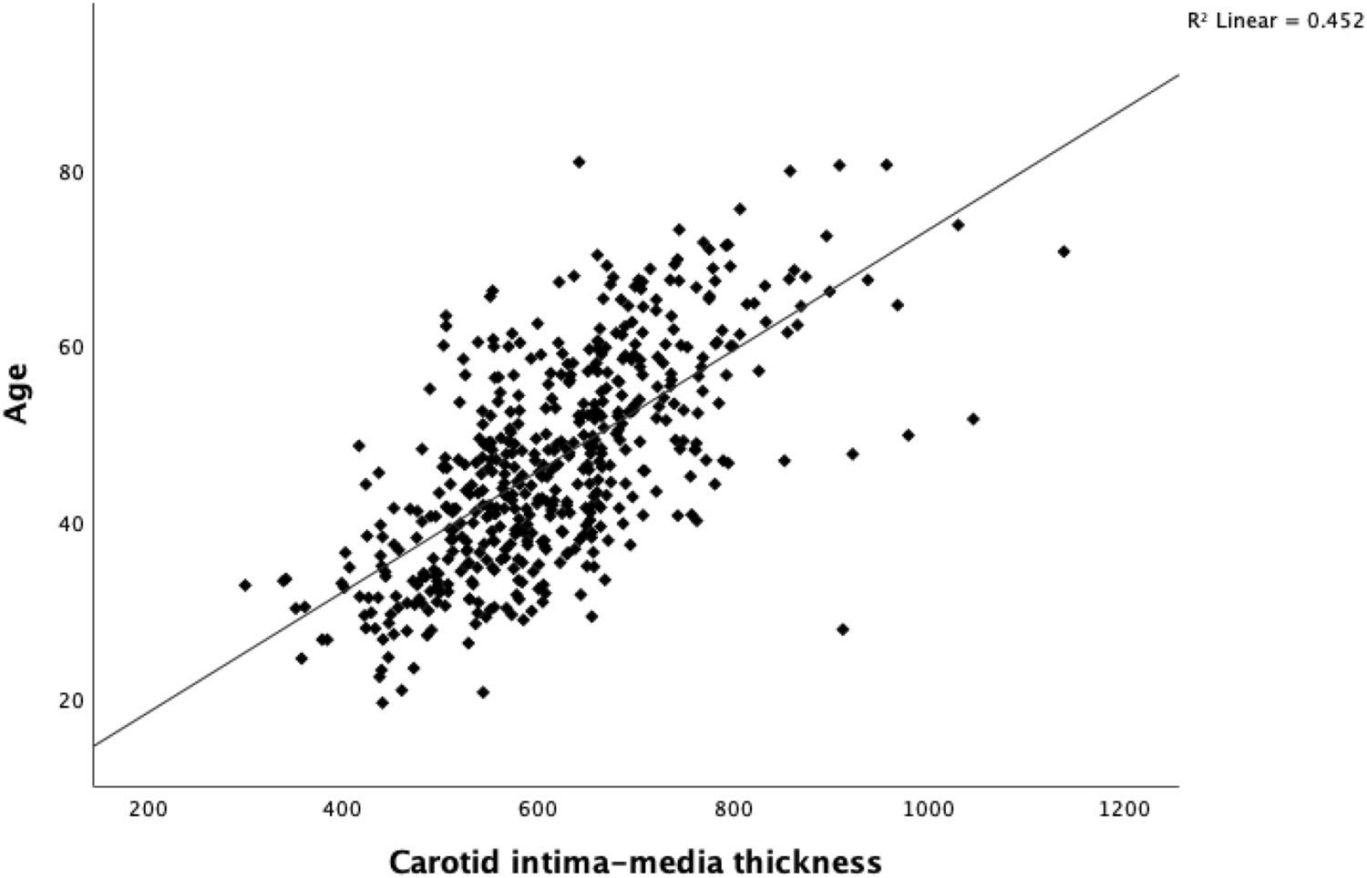
Carotid intima-media thickness (μm) by age in individuals with type 1 diabetes (*p* < 0.001)

Study participants had a median CIMT value of 606 μm (IQR 538–683 μm). CIMT did not correlate with HbA_1c_ at the time of the study visit (Fig. 1). Although there was a trend toward a correlation between the HbA_1c_-mean_overall_ and CIMT, it did not reach statistical significance (*p* = 0.063) (Fig. 2). Age was positively correlated with CIMT (Fig. 3).

### CIMT quartiles

Individuals were further divided into quartiles based on CIMT values. CIMT quartiles correlated with age and diabetes duration, while no correlation was observed between CIMT quartiles, sex, history of smoking, or BMI (Table 1).

Furthermore, systolic and diastolic blood pressure, use of anti-hypertensive drugs, use of renin–angiotensin–aldosterone system blockers correlated with CIMT quartiles. LDL cholesterol correlated inversely and the use of lipid-lowering drugs correlated positively with CIMT quartiles as well. No correlations were observed between CIMT quartiles and total cholesterol, HDL cholesterol, triglycerides, or hs-CRP (Table 1).

We further investigated the association between CIMT and diabetic kidney disease. Of note eGFR but not albuminuria correlated with CIMT quartiles (Table 1). Furthermore, a history of a cardiovascular event, stroke, coronary heart disease, peripheral vascular disease and retinal photocoagulation correlated with CIMT quartiles (Table 1).

HbA_1c_ levels at the time of the study did not correlate with CIMT quartiles in individuals with T1D. Finally, there was no correlation between the CIMT quartiles and HbA_1c_-mean_overall_ either (Table 1).

In multivariable linear regression analysis with CIMT as the dependent variable including HbA_1c_ or HbA_1c_-mean_overall_, age, sex, history of cardiovascular events, retinal photocoagulation, systolic blood pressure, the use of lipid-lowering drug, and estimated glomerular filtration rate, age was the only covariate that remained significantly associated with CIMT (Table 2). There was no observed correlation in the multivariable linear regression analysis, even after excluding individuals who had a history of cardiovascular events (Supplementary Table 1).

**Table 2:** Multivariable linear regression analysis of individuals with type 1 diabetes with carotid intima-media thickness as dependent variable and relevant risk factors, HbA_1c_ (Model 1) and HbA_1c_-mean_overall_ (Model 2) as independent variable

Carotid intima-media thickness				
Predictor	B	*p* value	Lower bound	Upper bound
*Model 1*			*95% Confidence Interval for B*	
Age	6.772	< 0.001	5.968	7.577
Sex	16.247	0.053	− 0.187	32.682
History of cardiovascular event	5.199	0.693	− 20.675	31.074
History of retinal photocoagulation	− 4.255	0.653	− 22.825	14.316
Systolic blood pressure	− 0.154	0.556	− 0.667	0.359
Lipid-lowering drug	− 1.085	0.909	− 19.675	17.506
Estimated GFR	0.110	0.491	− 0.205	0.425
HbA_1c_	2.072	0.551	− 4.749	8.894
*Model 2*			*95% confidence interval for B*	
Age	6.700	< 0.001	5.882	7.518
Sex	16.206	0.053	− 0.237	32.649
History of cardiovascular event	6.147	0.640	− 19.634	31.928
History of retinal photocoagulation	− 3.663	0.700	− 22.347	15.020
Systolic blood pressure	− 0.142	0.588	− 0.655	0.372
Lipid-lowering drug	0.187	0.984	− 18.419	18.794
Estimated GFR	0.115	0.474	− 0.200	0.430
HbA_1c_-mean_overall_	− 1.024	0.777	− 8.120	6.072

## Discussion

The main finding of our study was the lack of association between long-term blood glucose control and CIMT in individuals with T1D. The finding was surprising considering previous data showing associations between blood glucose control and CIMT in other cohorts of people with T1D. Only age was independently associated with CIMT.

Several variables were significantly correlated with CIMT after dividing the variable into quartiles. These factors were age, duration of diabetes, history of cardiovascular events, history of retinal photocoagulation, systolic blood pressure/anti-hypertensive medication, and eGFR. In contrast, LDL cholesterol or lipid-lowering medication showed a reverse relationship with CIMT, suggesting that older individuals with elevated CIMT and co-existing conditions were adequately treated with lipid-lowering medication.

Our multivariable linear regression analysis revealed that only age was independently associated with CIMT. The association between age and CIMT has been consistently observed across multiple studies involving different populations, such as the general population, and those with type 2 diabetes and chronic kidney disease, among others [18, 19].

At the time of the study visit, there was no observed correlation between CIMT and HbA_1c_ or HbA_1c_-mean_overall_. Although there was a tendency for HbA_1c_-mean_overall_ and CIMT to show an inverse correlation, this trend did not reach statistical significance (Fig. 2). It is possible that this trend may be attributed to the fact that older individuals with higher CIMT and longstanding diabetes may have established a more optimal glucose control, as opposed to younger patients who are still striving to achieve optimal diabetes control.

The DCCT/EDIC study investigated the impact of intensive insulin therapy on the progression of microvascular and macrovascular complications in individuals T1D. The results of the DCCT/EDIC study showed that intensive insulin therapy resulted in improved glucose control that was associated with slower progression of CIMT. The reason why our study did not yield similar results as the DCCT/EDIC, may be that our study was cross-sectional and had a retrospective design, while the DCCT/EDIC study was a randomized control trial. However, other factors may also contribute to the disparities. For instance, individuals in the DCCT/EDIC may have been more intensively managed, not only with respect to their blood glucose control, but also regarding lifestyle and pharmacologic treatment. However, our cohort presents real-time data on individuals with T1D in Finland. Additionally, the current epidemic of obesity and the co-occurrence of T1D and metabolic syndrome, known as hybrid diabetes, or presence of fatty liver disease may further complicate the relationship between CIMT and T1D in our study population [10, 20, 21].

Interestingly, no association was observed between CIMT and coronary artery events in the DCCT/EDIC, although intensive insulin therapy with subsequently improved glucose control was shown to reduce the risk of CVD in that trial [22]. Lastly, CIMT has not been shown to associate with cardiovascular events in individuals with T1D [6, 7, 23].

There is limited knowledge available regarding the relationship between T1D and CIMT. CIMT is a biomarker of atherosclerosis [1]. In the context of T1D, the pathogenesis of arterial disease is thought to involve calcification, endothelial dysfunction, and arterial stiffness. The role of atherosclerosis in arterial disease in these individuals is thought to be different compared to people without diabetes [24, 25]. Furthermore, individuals with the metabolic syndrome included in the study may partly explain the connection between CIMT and arterial disease more than hyperglycemia per se. This is an area for speculation and further investigation. It is possible that various cell-level factors may play a role in the advancement of CIMT in individuals with T1D. Some of these potential contributors include oxidative stress, chronic inflammation, the activation of the renin–angiotensin–aldosterone system, and the activation of the endoplasmic reticulum stress pathway [24, 26]. In our cross-sectional study, hs-CRP, a marker of chronic inflammation, did not correlate with CIMT. Overall, these findings highlight the complexity of the relationship between T1D, arterial disease, and atherosclerosis. Further research is needed to better understand the underlying mechanisms driving the progression of CIMT in T1D.

Our study has certain limitations that should be acknowledged. We did not collect data on short-term glucose control measures such as time in range (TIR) or variability obtained from continuous glucose monitoring systems (CGMS), which represents an area of interest for future studies to explore. No causal relationships can be explored given the cross-sectional nature of our study. However, it should be noted that blood glucose values from a ten-year period prior to the study visit were analyzed, which allowed for the assessment of cumulative and long-term blood glucose control.

Despite these limitations, our study has notable strengths that contribute to the understanding of CIMT in T1D. The cohort allowed robust analyses. Additionally, the use of standardized imaging and clinical assessments is a notable strength of our study, which enhances the reliability and validity of our results.

## Conclusion

We showed that long-term blood glucose control does not associate with CIMT in people with T1D. Age was the only factor independently associated with CIMT. This result differs from previous findings showing blood glucose control to be related to CIMT in T1D. Our findings, thus, suggest that there are other factors involved. We hope that our findings will pave the way for future studies that can further explore and shed light on the complex interplay of various factors in determining CIMT in T1D.

### Supplementary Information

Below is the link to the electronic supplementary material.

Supplementary file1 (DOCX 20 kb)

Supplementary file2 (DOCX 29 kb)

## Data Availability

Individual-level data of the study participants are not publicly available because of the restrictions due to the study consent provided by the participant at the time of data collection. Readers may, however, request collaboration with the authors to explore individual-level data by contacting the lead investigator.
