# Supplementary material for: No correlation between carotid intima-media thickness and long-term glycemic control in individuals with type 1 diabetes
Source: Acta Diabetol. 2023 Dec 10;61(4):441–9. doi: 10.1007/s00592-023-02211-y (PMC10963553; doi:10.1007/s00592-023-02211-y)
Supplement: Supplementary file 1 — Supplementary file1 (DOCX 20 kb) [file 592_2023_2211_MOESM1_ESM.docx]

**Supplementary Table 1** Multivariable linear regression analysis of individuals with type 1 diabetes and without history of cardiovascular event with carotid intima-media thickness as dependent variable and relevant risk factors, HbA_1c_ (Model 1) and HbA_1c_-mean_overall_ (Model 2) as independent variable

| **Carotid intima-media thickness** | | | | |
| --- | --- | --- | --- | --- |
| **Model 1** |  | | **95% Confidence Interval for B** | |
| **Predictor** | **B** | ***p* value** | **Lower Bound** | **Upper Bound** |
| Age | 6.684 | <0.001 | 5.803 | 7.565 |
| Sex | 11.923 | 0.183 | -5.639 | 29.484 |
| History of retinal photocoagulation | -6.580 | 0.517 | -26.509 | 13.349 |
| Systolic blood pressure | -0.027 | 0.927 | -0.599 | 0.546 |
| Lipid-lowering drug | 4.047 | 0.682 | -15.350 | 23.444 |
| Estimated glomerular filtration rate | 0.069 | 0.698 | -0.280 | 0.418 |
| HbA_1c_ | 1.244 | 0.738 | -6.059 | 8.547 |
|  | | | | |
| **Carotid intima-media thickness** | | | | |
| **Model 2** |  | | **95% Confidence Interval for B** | |
| **Predictor** | **B** | ***p* value** | **Lower Bound** | **Upper Bound** |
| Age | 6.645 | <0.001 | 5.743 | 7.547 |
| Sex | 11.999 | 0.181 | -5.604 | 29.602 |
| History of retinal photocoagulation | -6.430 | 0.528 | -26.445 | 13.586 |
| Systolic blood pressure | -0.020 | 0.945 | -0.593 | 0.553 |
| Lipid-lowering drug | 4.798 | 0.627 | -14.609 | 24.205 |
| Estimated glomerular filtration rate | 0.069 | 0.698 | -0.282 | 0.420 |
| HbA_1c_-mean_overall_ | -0.474 | 0.905 | -8.307 | 7.359 |
